# Associations of Depressive Symptoms, COVID-19-Related Stressors, and Coping Strategies. A Comparison Between Cities and Towns in Germany

**DOI:** 10.3389/fpsyt.2021.791312

**Published:** 2022-01-27

**Authors:** Caroline Meyer, Rayan El-Haj-Mohamad, Nadine Stammel, Annett Lotzin, Ingo Schäfer, Christine Knaevelsrud, Maria Böttche

**Affiliations:** ^1^Department of Clinical Psychological Intervention, Freie Universität Berlin, Berlin, Germany; ^2^Research Department, Center ÜBERLEBEN, Berlin, Germany; ^3^Department of Psychiatry and Psychotherapy, University Medical Center Hamburg-Eppendorf, Hamburg, Germany

**Keywords:** coronavirus, depression, restrictions, level of urbanization, pandemic stressors, mental health

## Abstract

**Background:**

The COVID-19 pandemic has led to a wide range of stressors related to depressive symptoms. Prevention measures like physical distancing have burdened the general population, especially in highly urbanized areas. However, little is known about the associations between pandemic-related stressors, coping strategies, and depressive symptoms in highly urbanized vs. less urbanized environments.

**Methods:**

Participants were recruited in a cross-sectional online survey in Germany. Propensity score matching yielded a matched sample of city (*n* = 453) and town (*n* = 453) inhabitants. Depressive symptoms, COVID-19-related stressors, and coping strategies were compared between cities and towns. Multiple regression analysis was performed to determine associations between pandemic-related stressors and depressive symptoms for the two groups separately.

**Results:**

City inhabitants showed significantly higher depression scores than town inhabitants (*t* = 2.11, df = 897.95, *p* = 0.035). Seven coping strategies were more often used by the city sample. Depressive symptoms were associated with “restricted physical social contact” and “difficult housing conditions” (adjusted *R*^2^= 0.19, *F*_[9,443]_ = 12.52, *p* < 0.001) in city inhabitants, and with “fear of infection” and “difficult housing conditions” (adjusted *R*^2^= 0.20, *F*_[9,443]_ = 13.50, *p* < 0.001) in town inhabitants.

**Limitations:**

The data were collected at the end of the first wave and represent a snapshot without causal inferences. Pandemic-related stressors were measured with a newly developed scale.

**Conclusion:**

Depressive symptoms, perceived stressors, and approach/avoidance coping strategies differed between city vs. town inhabitants. These differences should be considered in policy-making and mental health care.

## Introduction

The coronavirus disease 2019 (COVID-19) pandemic has affected the lives of hundreds of millions of people worldwide, changing their ways of living, working, and interacting with others. According to the latest figures provided by the World Health Organization (1), over 218 million people across the world have been infected and over 4.5 million people have died (1). To contain the spread of COVID-19, governments around the world have taken various non-pharmaceutical measures, including those that restrict physical social contact and movement. During the first wave of COVID-19 in Germany (02/2020 to 05/2020), these measures included contact restrictions (e.g., restricted personal contact), work-related restrictions (e.g., closure of shops and restaurants, working from home), restrictions in daily activity (e.g., reduced leisure activities), closure of educational institutions (e.g., schools, universities, and kindergartens), and border closures (2). These preventive measures have resulted in massive disruptions in economic systems and in people's personal lives, leading, among other problems, to job loss or reduced income (3). As a result of the variety of stressors caused by the pandemic and by preventive measures to contain the spread of infection, people have been confronted with a wide range of known risk factors for mental health problems, thus amplifying major mental health problems, and specifically depressive symptoms, worldwide (4–7). An increase in depressive and anxiety symptoms, as well as distress due to the pandemic, has also been reported in Germany (8, 9). As the virus spreads primarily through direct contact or airborne via droplets and aerosols, it spreads more easily in urbanized areas with a high population density (10). In general, people living in highly urbanized areas of high-income countries are more vulnerable to mental health problems than those living in less urbanized areas (11). Accordingly, the pandemic's impact on mental health has been found to be greater in highly urbanized and thus densely populated areas (12, 13). In a representative sample in Germany, a higher degree of urbanization was strongly associated with higher rates of mood disorders (14, 15). Moreover, studies have found that high population density, noise pollution, and light pollution in areas with a high degree of urbanization are responsible for higher levels of stress and consequently higher levels of affective disorders (16, 17). However, recent research has identified social stress, and specifically social isolation, as the most important risk factor for inhabitants of highly urbanized areas (18). During the pandemic, the long-term reduction of physical social contact, i.e., the social isolation, seems to have been associated with feelings of loneliness and disconnect from daily routine, leading to an increase in depressive and anxiety symptoms (19, 20). On the other hand, living in highly urbanized areas is not only a risk factor for inhabitant's mental health but also offers several advantages that can mitigate stressors due to the higher degree of urbanization. For instance, cities can provide better educational and professional opportunities, infrastructure, cultural opportunities, and health care (17, 21). However, many of these advantages and protective factors are no longer applicable due to the COVID-19 restrictions. In particular, measures to counteract social isolation were strongly restricted during lockdown, including cultural activities, social meeting points, public spaces and parks, restaurants or cafés, and other areas that enable and encourage social encounters (21).

Since many of the protective factors of life in cities are not present during the ongoing pandemic, it is additionally important to assess situational coping strategies that might buffer the pandemic-related stressors and might have an impact in terms of exacerbating or mitigating mental health problems [e.g. association between coping strategies and depression in older adults, (22)]. The most widely used measure to assess situational coping strategies is the Brief COPE (23, 24). According to a recent review (25), the most frequently used two-factor model classifies coping strategies into approach-related coping strategies, in which the individual actively approaches the stressor (e.g., active coping, acceptance), and avoidance-related coping strategies, in which the individual attempts to ignore the stressor or avoid its impacts (e.g., self-distraction, self-blame). Several studies have demonstrated an association between coping strategies and depressive symptoms during the pandemic. For instance, it was shown that approach-related coping strategies like positive reframing or active coping tend to be associated with lower levels of depressive symptoms (26–28). By contrast, avoidance-related coping strategies, such as self-distraction, behavioral disengagement, and self-blame, seem to be significantly associated with a higher degree of depressive symptoms (26–28). During the pandemic, the most frequently employed coping strategy is that of “acceptance” (27–29). Overall, the non-pharmaceutical measures that were implemented to control the number of infections during the first wave in Germany amplified the risk factors for depressive symptoms that are especially relevant in highly urbanized populations, such as social isolation, while simultaneously limiting the utilization of protective factors. Given the higher psychological burden in more urbanized areas and the restricted protective factors during the pandemic, a better understanding of pandemic-related stressors and protective factors such as coping strategies is needed. To develop adequate mental health response plans, it is crucial to understand the psychological consequences in areas with different population density and respective beneficial coping strategies. Therefore, the aim of this study was to compare the association of COVID-19-related stressors with depressive symptoms and coping mechanisms in relation to the level of urbanization in a German sample. In Germany, the majority of people (85%) live in urbanized areas such as towns (more than 5,000 inhabitants) or cities (more than 100,000 inhabitants). Due to Germany's high overall level of urbanization, living and working conditions in rural and suburban areas are strongly dependent on the infrastructure of the surrounding towns or cities, and the actual level of urbanization is therefore difficult to determine in suburbs and in rural areas. To minimize this heterogeneity, the present study focuses only on urban populations, with a town's population representing a moderate level of urbanization and population density and a city's population representing a high level of urbanization and population density.

In accordance with previous findings in the literature, the hypotheses of the study were threefold. First, we expected to find higher levels of depressive symptoms in the highly urbanized areas (city sample) compared to the moderately urbanized areas (town sample) during the COVID-19 pandemic. Second, we expected pandemic-related stressors (e.g., restricted physical social contact, problems with childcare, restricted access to resources) to show a differential association with depressive symptoms between the two samples. Third, we expected coping strategies to differ between the city and the town sample as a possible result of different levels of depressive symptoms.

## Methods

### Design and Sample

The cross-sectional study was part of a pan-European longitudinal study on psychopathology, pandemic-related stressors, and coping during the COVID-19 pandemic [30, study registry: https://doi.org/10.17605/OSF.IO/8XHYG]. An online survey was used to collect data from the general population in Germany between June and September 2020. Ethical approval was obtained from the Local Psychological Ethics Committee at the Center for Psychosocial Medicine (LPEK) at the University Medical Center Hamburg-Eppendorf (LPEK-0149).

Eligibility criteria included (1) minimum age of 18 years and (2) ability to understand and write in German. Prior to participation, all participants were informed about the aim of the study and provided informed consent. The link for the survey was sent via various networks to increase variability of the sample (e.g., social media, professional organizations, leisure and sports clubs). Participants received no compensation.

### Measures

In addition to the primary and secondary outcome measures, sociodemographic variables were assessed, including propensity characteristics (i.e., age, gender, COVID-19 infection, migration background, refugee background, general health status, partnership, number of children, household income, education) as well as the main variable for matching, i.e., self-reported residential area (city, suburb, town, rural area).

#### Depressive Symptoms

The Brief Patient Health Questionnaire (PHQ-9) assesses depressive symptoms during the last two weeks with nine items (30) rated on a 4-point Likert scale (0 = “not at all” to 3 = “nearly every day”). The overall score ranges from 0 to 27, with higher scores indicating more depressive symptoms. The measure has been validated in several populations (31, 32) and has shown excellent reliability (α = 0.86 to 0.91). The German version of the PHQ-9 is likewise well validated (32).

#### Pandemic Stressor Scale

The Pandemic Stressor Scale (33) assesses the perceived burden of COVID-19-related stressors during the last month with 30 items. Each item is rated on a 4-point Likert scale (0 = not at all burdened to 3 = strongly burdened), with an additional category “does not apply to me.”

The items are based on recently published research examining the burden of the COVID-19 pandemic. An exploratory factor analysis of a German sample yielded a nine-factor solution, which was cross-validated by a confirmatory factor analysis using the data of an Austrian sample of the ADJUST study (33).

Overall, nine COVID-19-related stressors, each containing up to five items, were identified: “Restricted physical social contact,” “Problems with childcare,” “Work-related problems,” “Fear of infection,” “Burden of infection,” “Restricted activity,” “Crisis management and communication,” “Restricted access to resources,” and “Difficult housing conditions.” Subscale scores were computed by calculating the average of the scores of the respective items. Before calculating the subscores, the category “Does not apply to me” was recoded into 0 (“Not at all burdened”). For details, see Appendix A.

#### Coping

The Brief COPE Inventory (23) is the short version of the COPE scale (34) and measures coping strategies on 14 two-item scales, with items rated on a 4-point Likert scale (1 = I haven't been doing this at all to 4 = I've been doing this a lot). The Brief COPE assesses situational coping responses to a specific stressor. In the current study, the COVID-19 pandemic was named as the specific stressor. According to Solberg et al. (25), the subscales of the Brief COPE are mostly categorized into two types of coping: approach coping styles (Use of emotional support, Use of instrumental support, Positive reframing, Acceptance, Active coping, Planning) and avoidance coping styles (Self-distraction, Denial, Substance use, Behavioral disengagement, Venting, Self-blame). The subscales humor and religion are not integrated in this dichotomy.

### Statistical Analyses

First, propensity score matching was performed to reduce the risk of selection bias due to different group sizes, but mainly to control for various confounding variables arising from the convenience sampling (i.e., non-randomized assignment of the two groups). The potential confounding variables used in the propensity score matching included age, gender, previous COVID-19 infection, migration status (own or parental migration), refugee status, subjective physical health status, partnership, having children, household income, and level of education. For propensity score matching on the groups of towns and cities, we used 1:1 matching on propensity scores with nearest neighbor matching without replacement, which is the most common form of matching (35, 36). To evaluate the balance of covariates, standardized mean differences (SMD) and level of significance were assessed before and after matching using *t*-tests for metric variables and *X*^2^ or Fisher's exact tests for categorical variables. An SMD of 0.1 or less indicates a negligible difference between two groups (37). A *t*-test was used to examine whether the groups differed with respect to the primary outcome of depressive symptoms.

Multiple regression analysis was performed separately for the city sample and the town sample to determine associations between pandemic-related stressors and depressive symptoms in each group. Finally, *t*-test analyses were conducted to determine whether the groups used different coping strategies.

Complete case analysis was used, as recommended for propensity score matching when data is missing at random (38). This method excludes all cases with missing data in the primary outcome or at least one of the covariates. All statistical analyses were performed using R4.0.2.

## Results

### Baseline Characteristics Before and After Matching

In total, *N* = 2,782 participants from all 16 Federal states of Germany participated in the cross-sectional online survey. We excluded participants who were not living in Germany at the time of the study (*n* = 30) or did not complete the Patient Health Questionnaire-9 (PHQ-9, *n* = 502), as this was the main outcome for the study. Given the aim of the present study, we excluded an additional n = 452 participants who lived in suburbs (*n* = 263) or rural areas (*n* = 189). The final sample before propensity score matching consisted of *N* = 1,798 participants, 1,319 of whom lived in a city (73.4%). Baseline characteristics before and after matching are shown in Table 1. Before matching, there were significant differences between city and town participants in terms of age (participants in towns were older), being in a partnership (more people in towns were living in a partnership), having children (more people in towns reported having children), and educational level (higher educational level in cities). The standardized mean difference of potential covariates ranged from−0.201 to 0.385.

**Table 1:** Covariates before and after propensity score matching.

	**Before propensity score matching**			**After propensity score matching**		
	**City** ***n*** **= 1319**	**Town** ***n*** **= 479**	***p***	**City** ***n*** **= 453**	**Town** ***n*** **= 453**	***p***
**Age** [M(SD)]	39.9 (12.4)	41.7 (12.5)	0.006^**^	41.5 (12.4)	41.73 (12.4)	0.750
**Female** (%)	930 (70.8)	342 (71.5)	0.795	305 (67.3)	322 (71.1)	0.250
**COVID-19 infection** (yes, %)	9 (0.7)	2 (0.4)	0.738	3 (0.7)	2 (0.4)	1
**Migration** (yes, %)	193 (14.6)	74 (15.4)	0.690	75 (16.6)	70 (15.5)	0.717
**Refugee** (yes, %)	7 (0.5)	1 (0.2)	0.613	0 (0.0)	0 (0.0)	-
**Health status** (%)			0.128			0.391
Very good	474 (35.9)	151 (31.5)		153 (33.8)	142 (31.3)	
Good	570 (43.2)	240 (50.1)		203 (44.8)	231 (51.0)	
Satisfactory	219 (16.6)	73 (15.2)		79 (17.4)	65 (14.3)	
Poor	50 (3.8)	14 (2.9)		16 (3.5)	14 (3.1)	
Very poor	6 (0.5)	1 (0.2)		2 (0.4)	1 (0.2)	
**Partnership** (yes, %)	903 (68.5)	367 (76.6)	<0.001^***^	343 (75.7)	350 (77.3)	0.638
**Children** (yes, %)	482 (36.5)	264 (55.1)	<0.001^***^	248 (54.7)	252 (55.6)	0.841
**Household income** (%)			0.110			0.964
Very low income	49 (3.8)	22 (4.8)		19 (4.2)	22 (4.9)	
Low income	96 (7.5)	22 (4.8)		25 (5.5)	22 (4.9)	
Medium income	543 (42.6)	180 (39.6)		186 (41.1)	180 (39.7)	
High income	375 (29.4)	139 (30.6)		135 (29.8)	138 (30.5)	
Very high income	211 (16.6)	91 (20.0)		88 (19.4)	91 (20.1)	
**Education** (%)			<0.001^***^			0.738
<10 years schooling	4 (0.3)	0 (0.0)		0 (0.0)	0 (0.0)	
≥10 years schooling	166 (12.6)	65 (13.6)		68 (15.0)	60 (13.2)	
Vocational studies	382 (29.0)	202 (42.2)		188 (41.5)	189 (41.7)	
Completed studies	767 (58.2)	212 (44.3)		197 (43.5)	204 (45.0)	

*Fisher's exact test was performed for the variables COVID-19 infection, refugee, health status, and education. Pearson's χ^2^ test was performed for gender, migration, partnership, children, and income. T-test was performed for age*.

** *p < 0.01*,

*** *p < 0.001*.

**Figure 1:**
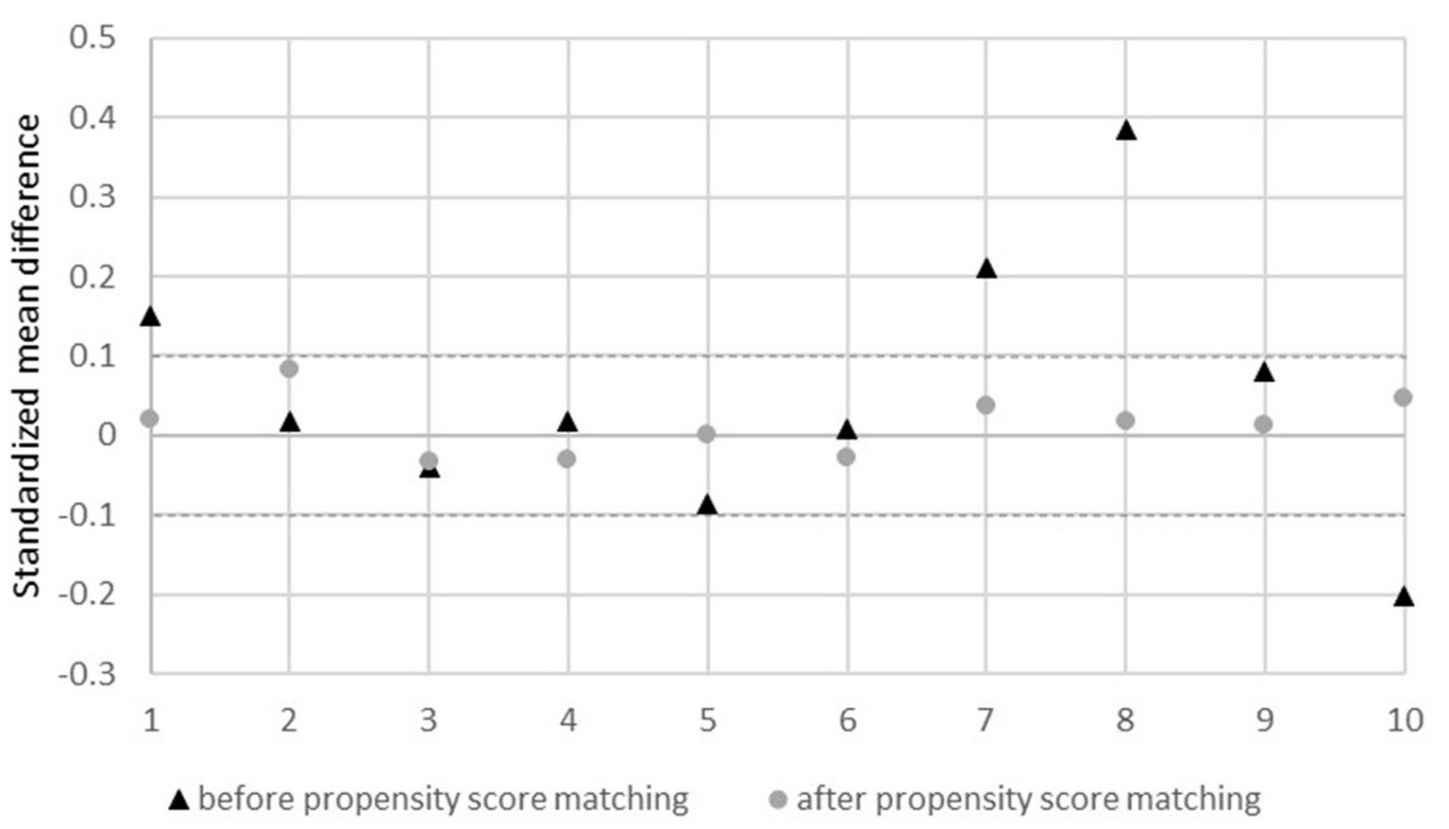
Standardized mean difference before and after propensity score matching. 1, age; 2, sex; 3, infection yes/no; 4, migration yes/no; 5, refugee yes/no; 6, health status; 7, partner yes/no; 8, children yes/no; 9, income; 10, educational level.

To evaluate the quality of our matched sample, we used both the *p*-value and the standardized mean difference as criteria. After propensity score matching, city and town samples did not differ substantially in all reported covariates (all *p* > 0.05, Table 1), and the standardized mean difference was within 0.1 (Figure 1). The matching process resulted in a total sample of *n* = 906 participants, with *n* = 453 in each group. In the matched sample, the age ranged from 18 to 78 years (*M* = 41.6, SD = 12.4) and the majority of participants were female (*n* = 627, 69.2%).

### Depressive Symptoms and COVID-19-Related Stressors in Cities and Towns

For all subsequent analyses, only the matched sample was considered. The city sample reported significantly higher levels of depressive symptoms compared to the town sample (*t* = 2.11, df = 897.95, *p* = 0.03, Table 2).

**Table 2:** Depressive symptoms measured by the PHQ-9 and stressors measured by the Pandemic Stressor Scale after propensity score matching.

	**City**		**Town**		***p***	**Cronbach's α**
	***n***	**M (SD)**	***n***	**M (SD)**		
**Depressive symptoms**						
PHQ-9	453	6.68 (5.13)	453	5.99 (4.72)	0.035^*^	0.86
**Pandemic Stressor Scale**						
Fear of infection	453	1.59 (0.73)	453	1.60 (0.73)	0.724	0.73
Restricted activities	453	1.56 (0.80)	453	1.46 (0.84)	0.063	0.72
Restricted physical social contact	453	1.51 (0.87)	453	1.43 (0.84)	0.163	0.85
Crisis management and communication	453	0.99 (0.75)	453	1.12 (0.79)	0.013^*^	0.71
Difficult housing conditions	453	0.65 (0.77)	453	0.52 (0.70)	0.005^**^	0.75
Work-related problems	453	0.64 (0.85)	453	0.77 (0.85)	0.024^*^	0.86
Problems with childcare	453	0.61 (1.08)	453	0.56 (1.00)	0.416	0.92
Restricted access to resources	453	0.60 (0.68)	453	0.60 (0.60)	0.849	0.62
Burden of infection	453	0.59 (0.79)	453	0.67 (0.84)	0.162	0.76

*PHQ-9, Patient Health Questionnaire-9. Differences in mean values between the subsamples were tested by t-test*.

* *p < 0.05*,

** *p < 0.01*.

Overall, people from cities and towns perceived similar COVID-19-related stressors. In total, the perceived stressors exerted low or moderate levels of burden in both samples (lowest burden *M* = 0.59, “burden of infection” in cities; highest burden *M* = 1.60, “fear of infection” in towns; range from 0 to 3). The following stressors were perceived to be the most stressful in both samples: problems with fear of infection (*M* = 1.59 in cities, *M* = 1.60 in towns), restricted activities (*M* = 1.56 in cities, *M* = 1.46 in towns), and restricted physical contact (*M* = 1.51 in cities, *M* = 1.43 in towns). Nevertheless, there were three significant differences between the town and city inhabitants: Participants in towns were more stressed because of work-related problems and “crisis management and communication” compared to those in cities. At the same time, participants living in cities were significantly more stressed due to the “difficult housing conditions” (Table 2).

### Associations Between COVID-19-Related Stressors and Depressive Symptoms in Cities and Towns

We conducted correlation analysis (Appendices B,C) and multiple regression analyses (Table 3) to examine the relationship between depressive symptoms and the pandemic stressor subscales for each sample separately. Correlates of depressive symptoms differed between the city and town samples (Table 3).

**Table 3:** Regression analysis of stressor subscales on depressive symptoms for city sample and town sample after propensity score matching.

	**City** **(***n*** = 453)**				**Town** **(***n*** = 453)**			
	***b***	***β***	**SE**	***p***	***b***	***β***	**SE**	***p-*** **value**
Intercept	3.01		0.64	<0.001^***^	1.02		0.63	0.106
Restricted physical social contact	1.31	0.22	0.32	<0.001^***^	0.25	0.04	0.30	0.404
Problems with childcare	−0.43	−0.09	0.24	0.072	0.18	0.04	0.23	0.425
Work-related problems	0.20	0.03	0.27	0.463	0.13	0.02	0.25	0.583
Fear of infection	0.51	0.07	0.36	0.156	1.66	0.26	0.33	<0.001^***^
Burden of infection	−0.21	−0.03	0.30	0.490	−0.26	−0.05	0.27	0.325
Restricted activities	−0.59	−0.09	0.34	0.081	0.40	0.07	0.29	0.166
Crisis management and communication	0.59	0.09	0.33	0.073	0.41	0.07	0.30	0.167
Restricted access to resources	0.17	0.02	0.37	0.642	−0.05	−0.01	0.37	0.892
Difficult housing conditions	2.10	0.31	0.36	<0.001^***^	1.76	0.26	0.34	<0.001^***^
Adjusted *R*^2^	0.19				0.20			

*** *p < 0.001*.

For the city sample, depressive symptoms were associated with “restricted physical social contact” and “difficult housing conditions” (adjusted *R*^2^= 0.19, *F*_[9,443]_ = 12.52, *p* < 0.001). For the town sample, depressive symptoms were associated with “fear of infection” and “difficult housing conditions” (adjusted *R*^2^ = 0.20, *F*_[9,443]_ = 13.50, *p* < 0.001).

### Coping Strategies in Cities and Towns

When comparing coping strategies between the two samples, the city inhabitants reported a higher use of seven out of fourteen coping strategies compared to those from towns. Participants living in cities reported significantly higher values on approach coping strategies (active coping, instrumental support, acceptance, emotional support) but also on avoidance coping strategies (venting, substance use) as well as the strategy “humor.” The most frequently used coping strategies in both samples were acceptance, self-distraction, and positive reframing (Table 4).

**Table 4:** Coping strategies by subsamples after propensity score matching and results of group comparison between city sample and town sample.

	**City** ***n*** **= 453 [M (SD)]**	**Town** ***n*** **= 453 [M (SD)]**	***p***	**Cronbach's α**
**Approach coping strategies**				
**Acceptance**	**3.69 (1.69)**	**3.44 (1.71)**	0.02^*^	**0.66**
**Positive Reframing**	**3.20 (1.88)**	**3.02 (1.81)**	**0.144**	**0.74**
Planning	3.17 (1.66)	3.03 (1.63)	0.218	0.53
Active Coping	2.92 (1.62)	2.55 (1.60)	<0.001^***^	0.60
Emotional Support	2.70 (1.72)	2.26 (1.68)	<0.001^***^	0.74
Instrumental Support	1.83 (1.59)	1.54 (1.56)	0.005^**^	0.82
**Avoidance coping strategies**				
**Self-Distraction**	**3.29 (1.63)**	**3.09 (1.59)**	**0.061**	**0.55**
Venting	1.95 (1.48)	1.74 (1.45)	0.028*	0.58
Behavioral Disengagement	1.03 (1.21)	0.91 (1.10)	0.115	0.32
Substance Use	0.81 (1.39)	0.51 (1.13)	<0.001^***^	0.92
Self-Blame	0.67 (1.24)	0.64 (1.22)	0.666	0.69
Denial	0.55 (1.09)	0.60 (1.07)	0.406	0.51
Humor	2.41 (1.72)	2.05 (1.60)	<0.001^***^	0.69
Religion	0.62 (1.29)	0.75 (1.43)	0.151	0.82

*Differences in mean values between the subsamples were tested by t-test; the three most frequently used coping strategies are printed in bold*.

* *p < 0.05*,

** *p < 0.01*,

*** *p < 0.001*.

## Discussion

The current study examined differences and similarities in depressive symptoms, COVID-19-related stressors, and coping strategies in city and town inhabitants in Germany. Through the use of propensity score matching, we were able to control for systematic differences between the two groups that may have resulted from convenience sampling. This allowed us to estimate, for the first time, a more precise representation of city and town inhabitants regarding the above-mentioned variables and shows the importance of the matched factors, as they varied significantly before matching. We found higher levels depressive symptoms in the city sample compared to the town sample in the matched samples, confirming previous results while controlling for several confounding factors. The relationship between pandemic related stressors and depressive symptoms differed between city and town inhabitants. Furthermore, city inhabitants reported a more frequent use of several coping strategies.

We found significantly higher levels of depressive symptoms in participants from cities compared to those from towns. Our results are in line with previous studies describing generally higher depressive symptoms in urban areas (11, 14). Moreover, they also correspond to recent studies that investigated populations in high-income countries during the pandemic and found a link between higher levels of urbanization and higher levels of mental distress (39, 40). This seems to reflect the effect of the non-pharmaceutical lockdown measures that were implemented to control the number of infections during the first wave of COVID-19 in Germany, which focused on contact restrictions and especially restricted time spent in public places both outdoors and indoors (2). These measures therefore amplified social isolation, being one of the main risk factors for depressive symptoms in city inhabitants (18). In accordance with this, the stressor “restricted physical social contact” was perceived as one of the most burdensome pandemic-related stressors by the city inhabitants. It was also strongly associated with depressive symptoms in the city sample but not in the town sample, indicating a potentially stronger impact of contact restrictions on depressive symptoms in more densely populated areas, though our cross-sectional design does not allow for causal inferences. At the same time, most of the benefits of living in cities (e.g., cultural activities, social meeting points) were eliminated due to the pandemic-specific restrictions. In contrast, access to outdoor spaces and a view of nature were found to be protective factors during the pandemic and are related to reduced levels of depressive and anxiety symptoms, especially under strict lockdown conditions (41). Both of these natural “buffers” are less available in cities.

Our study also aimed at a more differentiated understanding of possible factors influencing depressive symptoms in both cities and towns. Concerning stressors and coping strategies, we found both similarities and substantial differences. The findings on pandemic-specific stressors illustrate the extent to which the inhabitants of cities and towns felt stressed in various areas of everyday life and leisure during the pandemic. In the present study, only three significant differences emerged (“work-related problems” and “crisis management and communication” were higher in towns, “difficulties in housing conditions” were higher in cities), while the majority of pandemic-related stressors were perceived as equally burdensome in cities and towns. For both groups, the stressors perceived as the most burdensome were “fear of infection,” “restricted activities,” and “restricted physical social contact.” Studies have shown that infection-related stressors, i.e., fear of infecting others and loved ones, are perceived as highly stressful during the pandemic (42) due to the fact that the virus is life-threatening for people in high-risk groups [e.g., elderly, people with lung or heart diseases, (43)]. Furthermore, fear can also be explained by a lack of knowledge and by the unfamiliar and unpredictable new reality (44). The higher perceived stress with regard to restrictions of activities and physical social contacts appears to be self-evident due to the overall reported benefits of physical activity and social contacts (45).

“Work-related problems” as well as “crisis management and communication” were perceived as significantly more burdensome in towns. It is possible that people in cities can adapt more easily to crises due to a better infrastructure. This might, for example, include digitalization, better job opportunities in the case of job loss, better health care, and more services that offer support (46). These infrastructure advantages in cities could therefore mitigate the association between the aforementioned stressors and depressive symptoms. Previous research has already indicated an impact of media coverage on fears relating to COVID-19 (42). Garfin et al. (47) recommend using trustworthy and informative media and avoiding repetitive exposure to media with little new information. Especially in times of lack of knowledge, this is of high importance and could buffer the stressor “crisis management and communication.” In cities, “difficult housing conditions” were perceived as significantly more stressful. One explanation could be that the limited options in cities (e.g., small apartments, fewer social alternatives to seeing friends or family members, limited public spaces) were perceived as more burdensome.

The relationship between pandemic-related stressors and the severity of depressive symptoms illustrates that “difficult housing conditions” are associated with depressive symptoms in both samples. This is in line with previous findings suggesting that poor housing conditions, and especially limited space, are related to higher levels of depressive symptoms (48). In cities, the “restricted physical social contacts” were also significantly related to depressive symptoms. COVID-19 measures resulted in limited to no social contact over several months. As mentioned above, it can be assumed that these measures, especially in cities, aggravated a trend that has been found in previous studies. As previous findings show, people have begun to feel lonely during the pandemic, which is strongly correlated with depressive symptoms (19, 20). In towns, “fear of infection” was significantly related to severity of depressive symptoms. Due to a lesser social anonymity in towns, a potential fear of stigmatization as a result of an infection could explain this additional significant finding (49).

With regard to coping strategies, it was found that city inhabitants use seven of the examined 14 coping strategies significantly more often compared to town inhabitants (approach-related strategies i.e., active coping, acceptance, emotional and instrumental support; avoidance-related coping strategies, i.e., venting, substance use as well as the coping strategy “humor”). There were no significant differences in the other seven strategies. Recent studies have shown that “active coping,” “venting,” and “substance use” in particular are associated with depressive symptoms due to the pandemic (27). One explanation for why city inhabitants, on average, use more often strategies to cope with the pandemic could lie in the fact that depressive symptoms are higher in cities than in the towns, meaning that there is a greater need to use these strategies. However, research has shown that more frequent use of positive coping strategies might not be predictive of better positive adjustment over time (50). At this point, it is also important to mention that the rank order of the frequency of coping strategies used is the same in cities and towns. In both samples, “acceptance,” “positive reframing,” and “self-distraction” are used most frequently. This is in line with previous studies that also found “acceptance” and “self-distraction” to be among the most frequently used strategies during the pandemic (27–29). In a recent study, the coping strategy of “positive reframing” was the most beneficial in coping with depressive symptoms (27).

In our study, we found significant relationships between situational stressors and depressive symptoms even at an early stage of the pandemic. Presumably, these effects have intensified further over the course of the pandemic. The ongoing dilemma of lockdown and reopening has several implications, and the present findings emphasize that the level of urbanization has an impact on depressive symptoms as well as perceived COVID-19-related stressors.

## Limitations

The study findings should be interpreted in the light of several limitations. First, the data were collected in the period of June 2020 to September 2020. This period was at the end of the first wave of the pandemic, when infection rates were low and relatively few restrictions were in place in Germany. Second, as the data were cross-sectional, they represent a momentary snapshot of the situation without providing any information about the time course. Also, no statements can be made about representativeness as the sample was circumstantial and purposeful and the rate of return is unknown. However, different recruitment strategies were applied to increase the variability of the sample (e.g., social media, interest groups, companies). Third, pandemic-specific restrictions were measured using a newly developed instrument (33). Fourth, some of the subscales of the Brief COPE showed questionable or poor reliability scores in our study (Cronbach's Alpha: 0.53–0.92). This has also been reported in other studies (51, 52) and seems to be a general problem of the questionnaire, which is also reflected in the inconsistent factor structure of the Brief COPE (53). Fifth, although the propensity score matching has several advantages for examining the hypotheses and ensured comparability of our samples, the current dataset does not contain all participants and the representativeness of the two subsamples may have been altered especially in the city sample.

## Conclusion

Characteristics regarding depressive symptoms and coping strategies as well as the impact of pandemic-related stressors in cities and towns should be considered when addressing psychosocial support for vulnerable groups during and after the pandemic. Policy makers need to be aware of the special risks and needs in urban populations and should carefully evaluate the COVID-19-related measures taken in view of mental health costs and benefits. It seems to be important to investigate implications for different life circumstances and also to detect specific characteristics due to the level of urbanization. Future studies should therefore apply standardized measures of urbanization, e.g., by including population figures or other objective measures. Specifically, it becomes clear that restricted activities and physical social contact as well as housing conditions seem to be most burdensome in urban inhabitants. These stressors should receive special attention, both to better identify vulnerable people and to make future restrictions less stressful.

Long-term effects of the restrictions on mental health must be closely monitored, and mental health care offers need to be adapted to increased needs as early as possible.

This could be addressed in an easy and cost-effective manner by implementing low-threshold (online) interventions with instructions for self-help and self-care. In addition, longitudinal studies will be needed to differentiate between functional and dysfunctional coping strategies during and after the pandemic and to determine their effect on depressive symptoms. It is important to learn from this exceptional situation, to be able to give advice to vulnerable populations for the current situation and for potentially similar situations in the future.

## Data Availability Statement

The detailed sociodemographic information of the dataset does not fully protect the anonymity of the respondents. For this reason, the entire dataset cannot be made publicly available. However, excerpts of the data on a higher aggregation level can be provided upon justified request to the corresponding author.

## Ethics Statement

The studies involving human participants were reviewed and approved by Center for Psychosocial Medicine (LPEK) at the University Medical Center Hamburg-Eppendorf (LPEK-0149). The patients/participants provided their written informed consent to participate in this study.

## Author Contributions

AL designed the study in cooperation with the project steering committee formed by the representatives of the ESTSS countries [see (54)]. AL, MB, and RE-H-M were responsible for the data collection in Germany. CM, RE-H-M, and MB carried out the statistical analyses and drafted the manuscript. AL, IS, NS, and CK carefully revised the manuscript. All authors contributed to the article and approved the submitted version.

## Conflict of Interest

The authors declare that the research was conducted in the absence of any commercial or financial relationships that could be construed as a potential conflict of interest.

## Publisher's Note

All claims expressed in this article are solely those of the authors and do not necessarily represent those of their affiliated organizations, or those of the publisher, the editors and the reviewers. Any product that may be evaluated in this article, or claim that may be made by its manufacturer, is not guaranteed or endorsed by the publisher.
