# Supplementary material for: Associations of Depressive Symptoms, COVID-19-Related Stressors, and Coping Strategies. A Comparison Between Cities and Towns in Germany
Source: Front Psychiatry. 2022 Jan 27;12:791312. doi: 10.3389/fpsyt.2021.791312 (PMC8828998; doi:10.3389/fpsyt.2021.791312)
Supplement: Supplementary file 1 [file Data_Sheet_1.docx]

**Appendix A**

**Pandemic Stressor Scale (PSS)**

Please indicate how much the following things have burdened you due to the coronavirus pandemic within the last month.

| Stressor | | Not at all burdened | Somewhat burdened | Moderately burdened | Strongly burdened |
| --- | --- | --- | --- | --- | --- |
| Restricted physical social contact | |  |  |  |  |
|  | Social isolation | 0 | 1 | 2 | 3 |
|  | Restricted face-to-face contact with loved ones | 0 | 1 | 2 | 3 |
|  | Restricted face-to-face contact with others | 0 | 1 | 2 | 3 |
|  | Restricted physical closeness to loved ones | 0 | 1 | 2 | 3 |
| Problems with childcare | |  |  |  |  |
|  | Loss of childcare | 0 | 1 | 2 | 3 |
|  | Difficulties with combining work with childcare | 0 | 1 | 2 | 3 |
| Work-related problems | |  |  |  |  |
|  | Reduced working hours / fewer work orders | 0 | 1 | 2 | 3 |
|  | Not being able to work | 0 | 1 | 2 | 3 |
|  | (Threat of) income loss | 0 | 1 | 2 | 3 |
|  | (Threat of) job loss | 0 | 1 | 2 | 3 |
|  | Insufficient financial support by the government | 0 | 1 | 2 | 3 |
| Fear of infection | |  |  |  |  |
|  | Fear of getting infected with the coronavirus | 0 | 1 | 2 | 3 |
|  | Fear of infecting others with the coronavirus | 0 | 1 | 2 | 3 |
|  | Fear that loved ones will get infected with the coronavirus | 0 | 1 | 2 | 3 |
|  | Uncertainty about duration and risks of the coronavirus pandemic | 0 | 1 | 2 | 3 |
| Burden of infection | |  |  |  |  |
|  | My infection with the coronavirus | 0 | 1 | 2 | 3 |
|  | Infection of loved ones with the coronavirus | 0 | 1 | 2 | 3 |
|  | Death of a loved one due to the coronavirus infection | 0 | 1 | 2 | 3 |
| Restricted activities | |  |  |  |  |
|  | Restricted everyday activity (e.g., shopping) | 0 | 1 | 2 | 3 |
|  | Restricted leisure activity (e.g., restaurant visit) | 0 | 1 | 2 | 3 |
|  | Restricted private travel | 0 | 1 | 2 | 3 |
| Crisis management and communication | |  |  |  |  |
|  | Poor information from the government | 0 | 1 | 2 | 3 |
|  | Poor crisis management of the government | 0 | 1 | 2 | 3 |
|  | Media coverage of the coronavirus pandemic | 0 | 1 | 2 | 3 |
| Restricted access to resources | |  |  |  |  |
|  | Restricted access to goods, e.g., food, water, clothing | 0 | 1 | 2 | 3 |
|  | Restricted access to regular health care or medication | 0 | 1 | 2 | 3 |
|  | Insufficient capacity of the health care system for seriously ill people | 0 | 1 | 2 | 3 |
| Difficult housing conditions | |  |  |  |  |
|  | Restricted housing conditions (little space) | 0 | 1 | 2 | 3 |
|  | No place of retreat | 0 | 1 | 2 | 3 |
|  | Conflicts at home | 0 | 1 | 2 | 3 |

**Appendix B**

**Correlation matrix for stressors for city subsample after propensity score matching.**

|  | 1 | 2 | 3 | 4 | 5 | 6 | 7 | 8 | 9 | 10 |
| --- | --- | --- | --- | --- | --- | --- | --- | --- | --- | --- |
| 1 PHQ-9 | - |  |  |  |  |  |  |  |  |  |
| 2 Restricted physical social contact | .330^***^ | - |  |  |  |  |  |  |  |  |
| 3 Problems with childcare | .106^*^ | .094^*^ | - |  |  |  |  |  |  |  |
| 4 Work-related problems | .136^**^ | .170^***^ | 0.03 | - |  |  |  |  |  |  |
| 5 Fear of infection | .215^***^ | .396^***^ | 0.043 | .122^**^ | - |  |  |  |  |  |
| 6 Burden of infection | 0.07 | .172^***^ | -0.013 | .098^*^ | .403^***^ | - |  |  |  |  |
| 7 Restricted activities | .168^***^ | .540^***^ | 0.075 | .217^***^ | .194^***^ | 0.084 | - |  |  |  |
| 8 Crisis management and communication | .216^***^ | .286^***^ | .197^***^ | .212^***^ | .201^***^ | .124^**^ | .298^***^ | - |  |  |
| 9 Restricted access to resources | .177^***^ | .325^***^ | 0.073 | .161^***^ | .328^***^ | .211^***^ | .253^***^ | .373^***^ | - |  |
| 10 Difficult housing conditions | .361^***^ | .330^***^ | .502^***^ | .193^***^ | .191^***^ | 0.073 | .304^***^ | .268^***^ | .182^***^ | - |

*Note.* PHQ-9, Patient Health Questionnaire-9.

^*^p < .05. ^**^p <.01. ^***^p < .001.

**Appendix C**

**Correlation matrix for stressors for town subsample after propensity score matching.**

|  | 1 | 2 | 3 | 4 | 5 | 6 | 7 | 8 | 9 | 10 |
| --- | --- | --- | --- | --- | --- | --- | --- | --- | --- | --- |
| 1 PHQ-9 | - |  |  |  |  |  |  |  |  |  |
| 2 Restricted physical social contact | .277^***^ | - |  |  |  |  |  |  |  |  |
| 3 Problems with childcare | .182^***^ | .171^***^ | - |  |  |  |  |  |  |  |
| 4 Work-related problems | .121^**^ | .234^***^ | .115^*^ | - |  |  |  |  |  |  |
| 5 Fear of infection | .301^***^ | .299^***^ | 0.042 | 0.062 | - |  |  |  |  |  |
| 6 Burden of infection | .128^**^ | .163^***^ | .101^*^ | .119^*^ | .433^***^ | - |  |  |  |  |
| 7 Restricted activities | .141^**^ | .410^***^ | -0.025 | .239^***^ | -0.073 | 0.024 | - |  |  |  |
| 8 Crisis management and communication | .244^***^ | .404^***^ | .198^***^ | .176^***^ | .209^***^ | .118^*^ | .343^***^ | - |  |  |
| 9 Restricted access to resources | .139^**^ | .262^***^ | -0.002 | .113^*^ | .273^***^ | .164^***^ | .237^***^ | .343^***^ | - |  |
| 10 Difficult housing conditions | .363^***^ | .365^***^ | .439^***^ | .166^***^ | .154^**^ | .153^**^ | .171^***^ | .285^***^ | .108^*^ | - |

*Note.* PHQ-9, Patient Health Questionnaire-9.

^*^p < .05. ^**^p <.01. ^***^p < .001.
